# Challenges to HIV treatment adherence amongst adolescents in a low socio-economic setting in Cape Town

**DOI:** 10.4102/sajhivmed.v20i1.1002

**Published:** 2019-10-28

**Authors:** Brian E. van Wyk, Lee-Ann C. Davids

**Affiliations:** 1 School of Public Health, Faculty of Community and Health Sciences, University of the Western Cape, Cape Town, South Africa

**Keywords:** HIV, AIDS, adolescents, youth, adherence

## Abstract

**Background:**

Despite the successful rollout of anti-retroviral therapy (ART) and steep declines in HIV incidence in South Africa, this has not been the case for adolescents (10–19 years). Adolescents on HIV treatment have lower rates of viral load suppression and adherence compared to adults and children.

**Objectives:**

This article reports on the adherence challenges faced by adolescents receiving ART in a primary health care clinic in a low socio-economic urban setting in Cape Town.

**Method:**

An exploratory qualitative design was employed where data were collected through four focus group discussions with adolescents (*n* = 15) who received ART at a primary health care clinic in a low socio-economic urban setting in Cape Town and followed up with eight individual, semi-structured interviews with two adolescents from each focus group. Two key informant interviews were conducted with health workers at the clinic. Audio data were digitally recorded and transcribed verbatim. Data were analysed using content analysis.

**Results:**

School commitments, strained teacher–learner relationships, negative household dynamics and ill treatment by non-biological caregivers were reported as major barriers to adherence. In addition, poor service delivery, missing or misplaced files and long waiting times came under major criticism. Fear of unintended disclosure of HIV status, stigma and discrimination, treatment fatigue and having unstructured lives negatively influenced adherence. Having a strong social support system and having life goals and ambitions were motivators to remain adherent.

**Conclusion:**

This study highlighted the complexity of ART adherence in the midst of juggling school, home life and personal life goals and aspirations. Interventions to improve adherence should address psychosocial factors such as treatment fatigue, disclosure and family and household dynamics, in addition to streamlining service delivery between the school and clinic.

## Introduction

### Background

Globally, there are 2.1 million adolescents (10–19 years) estimated to be living with HIV in 2016, which accounts for 6% of all people living with HIV.^1^ The successful scale-up of antiretroviral therapy (ART) and Prevention of Mother To Child Transmission (PMTCT) programmes has led to the improved survival of perinatally infected children – who are now the ‘first generation’ of children with HIV entering adolescence.^2^ Despite improved access to ART and steep declines in HIV incidence and HIV-related mortality globally, HIV-related mortality amongst adolescents (15–19 years) has increased by about 50% between 2005 and 2012.^3,4^

In 2015, HIV was the second leading cause of mortality amongst adolescents globally and the leading cause of mortality in sub-Saharan Africa.^5^ The World Health Organization (WHO) argues that the reason for this statistic is partly because of insufficient prioritisation of adolescent health in national health programmes, poor provision of appropriate HIV testing and counselling (HCT) services and substandard follow-up care for adolescents who test HIV positive and who require ART.^4^ Further, it is widely reported that even when adolescents do access ART, adherence and retention in care and treatment outcomes are poorer compared to adults.^6^

Poor adherence to ART is one of the most significant challenges in ensuring patients achieve and maintain viral load suppression.^6^ Factors associated with poor adherence have been categorised as patient-related, structural, provider-related, disease-related, medication-related or psychological barriers.^7^ For adolescents, the transitional life period is characterised by physiological, psychological and intellectual development, which poses very unique challenges to ART adherence.^8^ The management of adolescents on ART, therefore, has to take cognisance of the complexity of biological and psychosocial changes which take place in the life of adolescents and its effects on adherence.^9,10,11^

### Research problem

It is posited that, amongst others, the reason for the poor adherence amongst adolescents (15–19 years) is because the transition from paediatric to adult HIV care programme is not well managed.^3^ However, there is a paucity of behavioural research to give insights into what the challenges and barriers are that adolescents with HIV face when receiving treatment in the adult ART programme.^10^

### Research aim

The aim of the study was to describe challenges to living with HIV and adherence to ART amongst school-going adolescents who receive ART at a public primary health care clinic in 2015–2016 in a low economic urban setting in the Western Cape province of South Africa.

## Methodology

### Study setting

This study took place at a municipal primary health care clinic in an urban residential area (township) in the greater Cape Town area, where the participants received HIV treatment services. The township is home to a predominantly African community.^12^ During the previous political regime (apartheid), this community was largely marginalised and exploited. The area spans 13.46 km^2^ and has a total population of 64 269, of which 96.3% are African people, 2.7% are mixed race people and 0.2% are white people. It is part of the Cape Town metro that carries the heaviest burden of HIV disease in the Western Cape with a prevalence of 5.2% in 2012.^12^

The clinic provides immunisation services, care for sick babies, TB treatment for drug susceptible and drug-resistant TB and HIV care for adults and children (wellness care and ART). It also provides antenatal care and family planning services. The clinic is open Monday to Friday from 07:30 to 16:30. The clinic is staffed by a pharmacist, two resident doctors, nurses, admin staff and a psychologist who comes to the clinic once a week. No dedicated adolescent services are provided.

The majority of the youth who access this facility come from the high school and primary school that are adjacent to the facility and utilised the clinic facilities before or after school. The number of adolescents who access the clinic for ART and HIV care is not known because routine HIV data are reported for paediatric (under 15 years) and adult (15 years and older) patients. The study was conducted from February to April 2016.

### Study design

An exploratory qualitative design^13^ was employed, because ART adherence is considered a very complex phenomenon and requires an in-depth understanding of the socio-cultural as well as the biological environment in which the behaviour occurs.

### Study population and sampling

Adolescents between 10 and 19 years who were registered to receive ART at the primary health care clinic in 2015–2016 and who were on ART for at least 6 months constituted the target study population and were subjected to purposive sampling. The inclusion criterion of 6 months on ART was chosen, because we wanted to explore participants’ adherence behaviour and experience on ART. Twenty-six participants were identified as eligible for inclusion to the study from their clinic files. However, only 15 participants were reachable and consented to participate in the study. Socio-demographic characteristics such as age and sex, clinical characteristics such as indications of adherence and initiation date of ART were extracted from patient folders to identify eligible participants. A summary of characteristics of adolescent participants is presented in Table 1. Participants’ adherence was identified as ‘poor’ or ‘good’ from the doctor’s notes in the patient folder. All participants in the study were perinatally infected.

**TABLE 1:** Characteristics of adolescent participants (*N* = 15).

Variables	Number of participants
**Age (years)**	
10–14	7
15–19	8
**Sex**	
Male	6
Female	9
**Years of ART**	
6 months–1 year	6
More than 1 year	9
**Reported adherence**	
Good	9
Poor	6

ART, antiretroviral therapy.

### Data collection

Data were collected through four focus group discussions (FGDs) and eight individual interviews in a language of the participants’ choice, that is, English or isiXhosa. All data were digitally recorded and transcribed verbatim. The researcher (LD) facilitated the FGDs and individual interviews and was assisted by an isiXhosa-speaking interpreter. isiXhosa transcriptions were translated to English. The researcher (LD) was a medical doctor, who worked in the HIV programme in City Health and conducted the current research towards her master’s degree.

The focus groups were divided according to gender and age as follows:

- Females, 10–14 years old – 3 participants
- Males, 10–14 years old – 4 participants
- Females, 15–19 years old – 6 participants
- Males, 15–19 years old – 2 participants.

Focus groups were age and gender aggregated, to allow free sharing within the group with a similar demographic or peer group. The FGDs were held in a meeting room in the facility.

Two participants from each FGD, who were identified to be willing to share more valuable insights, were approached for follow-up individual interviews. In the case of FGD 4, both participants agreed to be interviewed. The interviews and FGDs were semi-structured, with open-ended questions and prompts. An interview guide was compiled for this purpose in order to ensure standardisation across focus groups.

In addition, two key informant interviews were conducted with nurses who worked in the HIV programme in the clinic.

### Data analysis

The interviews were analysed manually making use of content analysis.^13^ Analysing the data started with reading and re-reading the transcripts several times. This was performed concurrently with reading the field notes, personal reflections and reading entries from my research diary.

On reading a transcript for the third time, the researcher (LD) made pencil notes in the margin of all the main issues that relate to adherence to treatment which came out from the text. The researcher was as inclusive as possible and also considered the things which were not being said such as suggestive statements and links between statements in different parts of the interview. Then, the list of all codes was transferred onto a separate page. In the next step, the researcher re-wrote the list of codes, but this time highlighting codes which were duplicated or emphasised by the participants. Similar codes were then grouped together, and in the last step, themes were developed. A consensus was reached between the researchers (LD and BVW) on the themes and codes.

### Trustworthiness

We followed Lincoln and Guba’s criteria for credibility, transferability, dependability and confirmability to enhance the trustworthiness of our study.^13^ The researchers held several meetings to debrief during data collection and analysis of interviews. Transcripts were shared amongst the researchers to check for quality and to check coding and formulation of themes. Disagreements were discussed until consensus on themes was reached. An independent person was used to transcribe the interviews and FGDs. Translation from isiXhosa to English was done by a first language speaker in isiXhosa, with master’s level qualification in public health.

### Ethical considerations

Ethics clearance for the study was provided by the University of the Western Cape Biomedical Research Ethics committee (Registration number: 15/7/254) and approved by City Health (ID number: 10537). All information was treated confidentially, and all participants’ anonymity maintained. Participation in the research was voluntary, and upon obtaining informed consent from all participants, and parents or guardians (if adolescent was younger than 18 years).

## Results

In this study, several barriers and one facilitator of adolescents’ ART adherence were identified. A presentation of these factors follows below.

### Barriers to adherence

The reported barriers to adherence were school, social, health services, treatment- and patient-related factors.

#### School factors

School factors such as school (work) commitment, communication with school teachers and negative teacher attitudes were found to play a deterring role in accessing the clinic, disclosure and adherence to ART. Participants often expressed feeling conflicted between school commitments and the need to attend clinic appointments. Even though there was a school adjacent to the clinic, many participants attended school elsewhere:

‘It would be nice for us to come at our own time, so that we do not have to miss our school work. That way we can be able to balance our life. Your school work doesn’t suffer because of the clinic appointments, and vice versa.’ (Group 4, male, 18)

In addition, the need to communicate attending regular clinic visits to teachers posed a significant barrier to attending regular clinic follow-ups as they feared unintended disclosure which may potentially lead to stigma and discrimination:

‘Okay my life orientation teacher is not a friendly person. She likes to shout, beat and [*is*] always angry. When she is angry, she says a lot of things out of anger; imagine now if you tell her about your status, and when she is angry she burst out in front of everyone. The best way is to keep this to myself.’ (Group 4, male, 16)

‘It does not sit well with me, because people will be suspicious, they will have questions about my frequent visits to the clinic. That does not make me feel right.’ (Group 4, male, 18)

#### Social factors

A lack of financial support was reported to have a negative impact on ART adherence, as participants often did not have money to pay for public transport to the clinic:

‘Oh … maybe I can use it [*financial support*] for a taxi fare to collect my medication.’ (Group 4, male, 18)

Participants cited negative relationships with non-biological caregivers as a barrier to adhering to ART with some orphans even saying they defaulted ART in an attempt to end their lives and be with their deceased biological parents:

‘Yoh, when I am feeling bad, I stop taking my pills. For example, when I have quarrelled with my mother, sometimes she says things that are not so nice. Like saying she never infected me. My mother is not my biological mother mos. My biological parents are dead. So when she talks like that I feel lonely and I feel bad, I stop taking the pills because I also want to die.’ (Group 4, male, 18)

Furthermore, adolescence is characterised by a desire to fit in. Some respondents reported feeling like outcasts amongst their immediate family because they were the only ones who were HIV positive:

‘I would not feel different from them [*if I was HIV negative*]; now I feel like an outcast. I am the only person with this thing [*HIV*] and I do not belong here. I feel like staying away from them … I wish I could stop taking medication [*ART*] and be like them.’ (Group 4, male, 18)

#### Health services factors

Long waiting times at the health facility were mentioned by all participants as a deterrent to adhering to clinic appointments (picking up medications), as these interfered with attending school that day:

‘… Sometimes if you come here at half past 8 and the doctor doesn’t attend you. … Doctors can make you wait for a very long time before they can attend you. You can come here at 8am and they only start to attend to you at 9. Mind you, you will go home at 4 that is why I get bored.’ (Group 1, female, 13)

In addition, participants feared that the flow of patients at this health facility may lead to unintended disclosure of their HIV status, because the area in which you waited in the clinic gave clear indication of the services you were coming for:

‘One time I came to fetch my medication and I saw my neighbour. Now she knows [*I am HIV positive*] because it’s obvious that people who wait this side are HIV positive.’ (Group 4, male, 16)

Missing and misplaced files also served as a major barrier to adhering to ART as adolescents would be told by clinic staff to return to the clinic on a different day to collect their medication:

‘I do go home without help, they keep losing my folder and sending me home and ask me to come back tomorrow. When I come again, they will say it is you again, and they are not keen to help me. Mind you I ran out of medication.’ (Group 3, female, 15)

Differential treatment and verbal abuse by health care workers towards patients who had defaulted or missed appointments deterred adolescents from returning to the clinic to re-start treatment:

Participant Is that I am scared because it’s been a while since I came to the clinic.

Facilitator And what are you scared of?

Participant Like the doctor is going to shout at me

Facilitator Anything else?

Participant The doctor will shout at me, and I will have to wait at the clinic since they will start with the non-defaulters. That makes me angry. (Group 4, male, 18)

A key informant admitted that often the unique attributes of adolescents were not completely understood by health care workers and that this impacted on service delivery to this group and resultant adherence. They were of the opinion that the health care system should be set up in such a way so as to focus on the unique needs of adolescents:

‘I don’t think we have systems in place yet because there is nothing that focuses on them (adolescents). They are just in another group of adults taking ARVs so there is nothing focusing specifically on assisting them so I would say we do not have a system that really supports them maybe if we could establish something that really focus on them because [*they*] are just dumped among the adults because they are just part of the whole group who is taking the ARVs.’ (Nurse)

#### Treatment-related factors

Treatment fatigue was mentioned as a profoundly significant barrier to adherence by all participants:

‘I sometimes feel that this thing that I have to take the pills every day does not sit well with me, I just get bored and feel like I could just throw them in the bin.’ (Group 1, female, 13)

‘Because it has been a while since I started taking the pills, I started when I was staying in Mandela at Worcester, but I was not used to them by then. I always ask mom and my sister, when I will ever stop taking these pills and my sister says, you will take them till you die. So that is why I do not like them.’ (Group 1, female, 13)

Participants in this study found the treatment routine associated with ART extremely rigid and were frustrated by the fact that apparently no leniency was allowed:

‘Yah, I also miss them sometimes. I am a playful person. So at times I miss my time and I do not want to take them after that time because I do not know if they will have negative effects after.’ (Group 4, male, 16)

#### Patient-related factors

The dynamics of disclosure played a pivotal role in ART adherence amongst these participants. The majority of participants preferred selective disclosure where they chose to disclose their HIV status to some people but not to others. Many participants feared rejection, stigma and discrimination if their status is known. Therefore, participants chose not to disclose their HIV status or that they are on treatment to friends, even when they sleep over:

‘I don’t want to disclose my [*HIV*] status to my neighbour or to my friends. I want to disclose my status only to my family.’ (Group 3, female, 17)

Furthermore, some of the younger participants did not disclose their status because their parents forbade them to do so. This may be because if an adolescent discloses their HIV status they may also indirectly be disclosing their parent’s HIV status:

‘Because my mother told me that I must not tell anyone [*about my HIV status*].’ (Group 1, female, 10)

‘They [*my parents*] prohibit us from talking about it [*our HIV status*].’ (Group 2, male, 14)

Many participants felt that no harm would come to them if they miss taking their medication on occasions. They would rather have fun with friends and have unplanned sleepovers following parties than come home to take their medication. The participants reported that they do not make provision to take their medication with them in the event of social functions:

‘There are times when there is a party somewhere and my friends will be attending and I also have to go with them. In those instances we come home the following day, and I will miss my pills. Those gatherings are fun, I can’t leave fun mos.’ (Group 4, male, 18)

‘Sometimes, like holiday like December like its few party. And so like if I was at a party with friends, like maybe I am whatever place with my friends but my friends did not know I was HIV positive. So when it was 9 o’clock it was difficult to just leave. So I would just think argh, so what if I don’t take them, nothing will change I will just take them on another day.’ (Group 3, female, 15)

Some participants reported feelings of being alone and not normal or dirty because they were HIV positive and have to take medication:

‘To be like a normal person, when I am taking these ARVs I don’t feel like a normal person because everybody does not drink these pills. I feel like I am the only one here that drinks these pills.’ (14-year-old male)

‘… It is me alone at my home that is drinking the medication and that it makes me … it makes me feel very lonely.’ (14-year-old male)

‘Not now like last year a lot because I feel bored and I feel no future. I am dirty I feel like I take the pills to the toilet and flush [*the pills*].’ (Group 3, female, 15)

### Facilitators of adherence

Receiving social support from family members, particularly siblings, and friends encouraged participants to remain adherent to their ART:

‘Okay we are four, it’s me, my mom, and my two sisters. But I am close to my mom. Me and my big sister we quarrel a lot, even out of nothing. She knows about my treatment but when we have fights she doesn’t say anything about it, even when I am tired of taking it they encourage me to continue. They always check if I take my treatment and they will notice that I am not taking it. We are such a close family but I am closer with my mother.’ (Group 4, male, 16)

‘Yah because I feel like I am free, even when he [*my friend*] visits my place or I visit his place I feel free to take my medication. When I am sleeping over at his place, when I say I am going to take my pills he understands and even remind me some days.’ (Group 4, male, 18)

## Discussion

The findings of our study indicate three extended themes, namely the conflict between the school and clinic, the need for HIV-competent households and adolescent-friendly HIV services.

### Conflict between school and clinic

The importance of keeping adolescents living with HIV in school has been expressed by researchers and social activists, because education reduces the vulnerability of girls and instils hopefulness for the future in all adolescents.^14,15,16^ It is thus concerning that this study reports conflicted commitments to school attendance and making clinic appointments. In addition, the need to communicate attending regular clinic visits to teachers posed a significant barrier to attending regular clinic follow-ups as they feared unintended disclosure which may potentially lead to stigma and discrimination. The findings of the study are in keeping with the literature which suggests that mainstream schooling may not necessarily always have a positive impact on adherence. In this study, the routine of schooling also made clinic visits difficult and some participants felt that their frequent absences may lead to unintended disclosure of their HIV status. It is well documented that if adolescents disclose their status to a trustworthy person or people, they are more likely to receive help in the form of knowledge and resources to help them cope with a HIV diagnosis and to access and remain in HIV care.^4,5^ We recommend that educators should be sensitised – HIV competent – to handle HIV disclosure of learners with sensitivity and understanding.

### HIV-competent households

A lack of financial support and negative household dynamics were found to have a negative impact on ART adherence. The findings of the study are thus in keeping with the literature on the positive effects of financial support and/or income security of households on adherence amongst adolescents.^4,15^ In our enquiry, negative household dynamics had a detrimental effect on reported ART adherence. Some participants reported feeling like outcasts in their family as a result of being the only family member who was HIV positive and on treatment. This was also the reason they gave for sometimes feeling like ending their lives by not taking their medication. The findings are in keeping with the literature that identifies parenting and family dynamics as playing a pivotal role in facilitating adherence to ART.^4,17,18^

### Adolescent-friendly HIV services

Our study found health systems barriers to ART adherence in the form of long waiting times and missing or misplaced files, and the risk of inadvertent disclosure of HIV status. These barriers have been reported previously and are not unique to adolescents.^19,20,21,22^ Literature recommends the integration of youth-friendly services such as evening clinics, adolescent clinic days and youth-friendly waiting areas within ART programmes.^23^

### Reconciling adolescence and taking medicine

All participants reported treatment fatigue as a barrier to adherence. Participants in this study were frustrated that the treatment routine was extremely rigid and that no leniency was allowed. This is in keeping with the literature that identified treatment fatigue as a major factor impacting on older adolescents’ ability to remain adherent to a treatment regimen.^24^ There was a suggestion from the older adolescents in this study that having ART-free weekends would greatly improve their adherence.^25^

The nature of disclosure remains a pivotal role in ART adherence as also reported in this study. The participants in this study preferred selective disclosure where they chose to disclose their HIV status to some people but not to others, which is in line with the current WHO guidelines.^4^ Our study found that participants reported improved adherence once their HIV status had been disclosed to them and they have disclosed their HIV status to supportive friends. These findings are congruent with the literature that says if adolescents disclose their status to a trustworthy person or people, they are more likely to receive help in the form of knowledge and resources to help them cope with a HIV diagnosis and to access and remain in HIV care.^4,26^

Our study found indications of depressive symptoms through participants reporting feelings of being alone and dirty (self-stigma) and contemplating ending their lives. This finding is consistent with the literature that reports higher prevalence of emotional and behavioural problems amongst adolescents living with HIV compared to other high-risk groups.^27,28^ A meta-analysis reports that patients with depressive symptoms were 42% less likely to achieve optimal adherence to ART regimen.^29^

## Conclusion

This study highlighted the complexity of ART adherence in the midst of juggling school, home life and personal life goals and aspirations amongst a sample of school-going adolescents on ART in a low socio-economic urban township in the Western Cape. Whereas it was encouraging that the adolescents were all in school, the lack of collaboration between the education sector and the health sector in the interest of the adolescent and learner’s adherence to treatment is of great concern. More research is needed to unpack the complexity of this intersection and to develop guidelines for integration and collaboration between these two sectors that could be mirrored at implementation level between the school and the clinic.

Interventions to improve adherence should address psychosocial factors such as treatment fatigue, depressive symptoms, disclosure and family and household dynamics amongst adolescents. In other facilities in the Western Cape province, family clinics and youth clubs have been implemented to boost adherence. Future studies should seek to explore how these initiatives could be mainstreamed across all facilities in the province.

### Limitations of the study

This study is of limited scope, because it was conducted towards the fulfilment of requirements for a master’s degree with coursework. The sample size was relatively small, and data saturation was not achieved. The sample was drawn from adolescents who were still in care; therefore, some measure of adherence was present. Valuable lessons about adherence challenges might be missed because those who defaulted from treatment were not included in the study.
